# Complete genome sequence of a plant growth-promoting *Methylorubrum aminovorans* MtTm-13

**DOI:** 10.1128/mra.00224-26

**Published:** 2026-06-22

**Authors:** Pragadeesh Ayyamuthu Rajarathinam Uma, Sandhya Namadara, Abishagu Arulanandar, Prakash Kolanchi, Dinesh Antony Vedhamuthu, Nanthitha Ashok, Jadav Pavan, Alisha Farheen, Senthilkumar Murugaiyan, Vidya de Gannes

**Affiliations:** 1 Department of Agricultural Microbiology, Tamil Nadu Agricultural University 29918, , Coimbatore, Tamil Nadu, India; 2 Department of Agricultural Entomology, Tamil Nadu Agricultural University 29918, Coimbatore, Tamil Nadu, India; 3 Department of Food Production, The University of the West Indies 37612, Saint Augustine, Trinidad and Tobago; University of Wisconsin-Madison, Madison, Wisconsin, USA

**Keywords:** *Methylorubrum aminovorans*, genome, plant growth-promoting

## Abstract

We report the complete genome of plant growth-promoting *Methylorubrum aminovorans* MtTm-13 (5,231,310 bp, 69% GC, 4,660 protein-coding genes) isolated from the tomato phyllosphere.

## ANNOUNCEMENT

The genus *Methylorubrum*, formerly classified under *Methylobacterium*, is facultative methylotrophic Alphaproteobacteria (1). These are pink-pigmented, gram-negative, ubiquitous in nature and possess the ability to utilize single-carbon compounds or methyl groups as the sole carbon source (2). They exhibit plant growth-promoting traits, including hormone modulation, mineral solubilization, and the production of 1-aminocyclopropane-1-carboxylate deaminase and siderophores, which aid plant growth and reduce adverse effects of abiotic stress (3). Strain MtTm-13, a stress-tolerant plant growth-promoting bacterium (4, 5), was isolated from leaves of a tomato plant at the Tamil Nadu Agricultural University, Coimbatore (11.0069° N, 76.9309° E), India. The leaf sample was surface-sterilized with 2% sodium hypochlorite and rinsed with sterile distilled water three times. The sterilized leaf sections were then imprinted onto ammonia mineral salt (AMS) agar containing 0.5% methanol and cycloheximide (10 μg/mL) and incubated at 28 ± 2°C for 7 days, until a distinct pink colony appeared. Whole-genome sequencing was performed to elucidate the genetic basis of its plant growth-promoting potential.

Genomic DNA was isolated from a single purified colony, grown in AMS broth at 30°C for 7 days under shaking conditions, using the QIAGEN Genomic-tip 20/G kit (QIAGEN, Hilden, Germany), following the manufacturer’s instructions (6, 7). DNA quantity and integrity were evaluated using a Qubit 4 fluorometer (Thermo Fisher Scientific, Waltham, MA, USA) and agarose gel electrophoresis. The purified DNA was then submitted to ONEOMICS Private Limited (Tiruchirappalli, India) for whole-genome sequencing. Illumina sequencing libraries were prepared with the TruSeq DNA PCR-Free Kit (Illumina, USA), following the manufacturer’s instructions. Briefly, genomic DNA was fragmented, end-repaired, A-tailed, size-selected, and ligated with indexed adapters. Library concentrations were quantified using a QuantStudio Real-Time PCR System (Applied Biosystems, USA), and fragment size profiles were assessed using an Agilent 2100 Bioanalyzer (Agilent Technologies, USA) (8). Sequencing was carried out on the Illumina NovaSeq X platform, generating 64,041,602 paired-end reads (2 × 150 bp). No modifications were made to the standard protocol.

Raw reads were quality-checked using FastQC v0.10.1 and trimmed with Trimmomatic v0.39 (9, 10). Genome assembly was produced using Shovill v1.4.2 (11), and the draft assembly was subsequently scaffolded with RagTag v2.1.0 (12) using reference genome OZ375356.1, selected based on 16S rRNA gene sequence analysis. Assembly quality was assessed using QUAST v5.3.0 (13). The genome spans 5,231,310 bp with a GC content of 69% and sequencing depth of 1,332.75× (Table 1). Genome completeness was estimated at 98.59%, using BUSCO v5 with the bacteria_odb10 data set (14, 15). Functional annotation using PGAP v6.10 (16) revealed genes associated with plant growth promotion and stress tolerance, including tryptophan synthase subunits (*trpA* and *trpB*) for IAA biosynthesis, phosphate transport genes (*phoB* and *pstS*), siderophore receptors (*fhuF*) for iron acquisition, and catalases and superoxide dismutases. Taxonomic classification was carried out using the GTDB-Tk v1.7.0 against GTDB reference set (17, 18). Unless noted, all software was used with default parameters.

**TABLE 1:** Detailed information of draft genome

Feature	Value
Genome size	5,231,310 bp
Number of chromosomes	1
Contig N50	5,231,310 bp
Contig L50	1
GC content (%)	69
Genome coverage	1,332.75×
Assembly level	Chromosome
BUSCO completeness	98.59%
SRA	SRS27774344
GenBank Assembly	CM143384

## Data Availability

The complete genome sequence of *Methylorubrum aminovorans* MtTm-13 has been deposited in GenBank under the accession number CM143384. The raw sequencing reads are available under BioProject accession number PRJNA1403065, and the BioSample accession number is SAMN54618850. All sequencing data produced in this study are accessible through the Sequence Read Archive (SRA) under accession SRS27774344. Additionally, the assembled genome sequence is available in GenBank under accession number GCA054962035.1.
